# Based on disulfidptosis-related glycolytic genes to construct a signature for predicting prognosis and immune infiltration analysis of hepatocellular carcinoma

**DOI:** 10.3389/fimmu.2023.1204338

**Published:** 2023-08-23

**Authors:** Zhijian Wang, Xuenuo Chen, Jia Zhang, Xuanxin Chen, Jiayi Peng, Wenxiang Huang

**Affiliations:** ^1^ Department of General Practice, The First Affiliated Hospital of Chongqing Medical University, Chongqing, China; ^2^ Department of Infectious Disease, The First Affiliated Hospital of Chongqing Medical University, Chongqing, China; ^3^ Department of Geriatrics, The First Affiliated Hospital of Chongqing Medical University, Chongqing, China

**Keywords:** hepatocellular carcinoma (HCC), disulfidptosis, glycolysis, subtype, prognostic signature, tumor microenvironment, SLCO1B1

## Abstract

**Background:**

Hepatocellular carcinoma (HCC) comprises several distinct molecular subtypes with varying prognostic implications. However, a comprehensive analysis of a prognostic signature for HCC based on molecular subtypes related to disulfidptosis and glycolysis, as well as associated metabolomics and the immune microenvironment, is yet to be fully explored.

**Methods:**

Based on the differences in the expression of disulfide-related glycolytic genes (DRGGs), patients with HCC were divided into different subtypes by consensus clustering. Establish and verify a risk prognosis signature. Finally, the expression level of the key gene SLCO1B1 in the signature was evaluated using immunohistochemistry (IHC) and quantitative real-time PCR (qRT-PCR) in HCC. The association between this gene and immune cells was explored using multiplex immunofluorescence. The biological functions of the cell counting kit-8, wound healing, and colony formation assays were studied.

**Results:**

Different subtypes of patients have specific clinicopathological features, prognosis and immune microenvironment. We identified seven valuable genes and constructed a risk-prognosis signature. Analysis of the risk score revealed that compared to the high-risk group, the low-risk group had a better prognosis, higher immune scores, and more abundant immune-related pathways, consistent with the tumor subtypes. Furthermore, IHC and qRT-PCR analyses showed decreased expression of SLCO1B1 in HCC tissues. Functional experiments revealed that SLCO1B1 overexpression inhibited the proliferation, migration, and invasion of HCC cells.

**Conclusion:**

We developed a prognostic signature that can assist clinicians in predicting the overall survival of patients with HCC and provides a reference value for targeted therapy.

## 1 Introduction

Primary liver cancer is a prevalent malignancy worldwide and the fourth leading cause of cancer-related deaths. The highest incidences were observed in East Asia, Southeast Asia, and North Africa (1). Hepatocellular carcinoma (HCC) accounts for approximately 80% of all primary HCCs (2). HCC typically arises from the progression of metabolic liver disease or viral hepatitis B and C infections (2). Numerous genetic mutations build up during the development of HCC, including TP53 mutations in hepatitis B-associated HCC and CTNNB1 and TERT mutations in HCC associated with alcoholic liver disease (3). Owing to its insidious onset, HCC is usually diagnosed at an advanced stage when surgical intervention is not feasible. Current treatment options for patients with advanced HCC include radiotherapy, immunotherapy, and targeted therapy. However, their efficacy is often limited by drug resistance (4, 5). Therefore, identifying new tumor markers is crucial for improving HCC-targeted therapies.

Recent studies have revealed that the accumulation of intracellular disulfide induces a stress response leading to disulfidptosis, a novel form of programmed cell death (6). Cancer cells typically rely on the amino acid transporter protein SLC7A11 to transport cystine intracellularly and regulate tumor growth. However, cystine is a disulfide that may have cytotoxic effects. To balance this, cells rapidly convert toxic disulfide to other non-toxic molecules using nicotinamide adenine dinucleotide phosphate (NADPH) (7). NADPH is mainly produced by glucose metabolism, and in cases where tumor cells are deficient in glucose, it can trigger disulfidptosis in tumor cells, which in turn inhibits tumor growth. However, this process does not cause cytotoxic to normal tissues (8). Since the introduction of the concept of disulfidptosis, it has attracted considerable attention from the medical community, particularly in the field of tumor treatment (9). Therefore, understanding the state of disulfidptosis in different patients with HCC is valuable for exploring targeted therapies for HCC.

Glycolysis is a method of metabolic reprogramming in tumor cells and was initially identified during the study of HCC. The hallmark feature is that tumor cells use glycolysis as the main energy source, even when mitochondria function normally and oxygen is available, leading to a significant increase in the cellular uptake of glucose and lactic acid production (10, 11). In addition to playing a crucial role in tumor proliferation, metastasis, and invasion, glycolysis partly explains the development of resistance to sorafenib in HCC (12, 13). Targeting glycolysis holds promise for improving drug resistance and is a potential therapeutic target for HCC.

**Figure 1:**
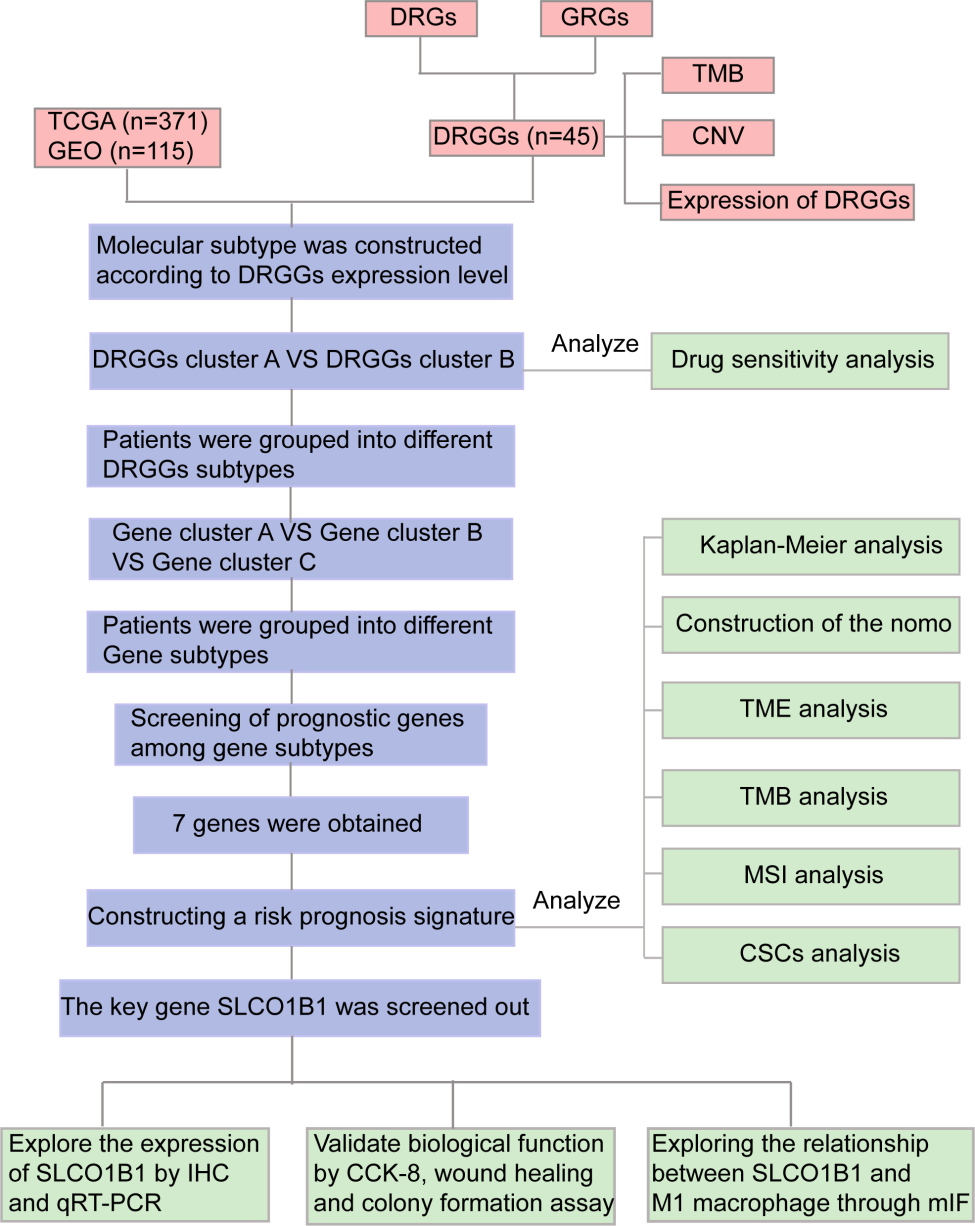
Flow chart of our study. DRGs, disulfidptosis related genes; GRGs, glycolysis related genes; DRGGs, disulfidptosis related glycolytic genes; TCGA, The Cancer Genome Atlas; GEO, Gene Expression Omnibus; TMB, tumor mutational burden; CNV, copy number variation; GSEA, Gene Set Enrichment Analyses; TME, tumor microenvironment; MSI, microsatellite instability; CSCs, cancer stem cells; IHC, immunohistochemical; DEGs, differentially expressed genes; CCK-8, Cell counting kit-8; mIF, multiplex immunofluorescence.

As two important biological processes in tumors, the relationship between disulfidptosis and glycolysis has received considerable attention. Although the concept of disulfidptosis is relatively new, studies on sulfur metabolism in tumors have been reported. Researchers have proposed that sulfur-containing compounds from garlic inhibit the proliferation of HCC cell lines, a process closely associated with the highly reactive sulfane sulfur (14, 15). In humans, sulfur-containing amino acids, such as cysteine, and sulfur-containing proteins, such as glutathione, are metabolized to produce sulfane sulfur, which has both anti-cancer and pro-cancer effects. However, the mechanism of action is unclear, and we speculate that there may be a link between disulfidopathy and the therapeutic outcomes of sulfur-containing compounds (14, 16–18). Additionally, sulfur-containing amino acids and disulfide proteomics have great potential for regulating glycolysis (19, 20). These studies inspired us to further explore the association between disulfidroptosis and glycolysis in patients with HCC and healthy individuals to guide targeted therapy and prognosis of HCC. In this study, we developed a prognostic signature by combining disulfide-related genes (DRGs) and glycolysis-related genes (GRGs) to predict the prognosis of HCC patients. The flowchart in **Figure 1** illustrates how this study was conducted.

## 2 Materials and methods

### 2.1 Collation and collection of data

First, we downloaded clinicopathological information, gene expression matrix data, and somatic mutation data of patients with HCC from The Cancer Genome Atlas (TCGA) database. Another set of data containing the survival information of patients with HCC was downloaded from the Gene Expression Omnibus (GEO) database, and joint analysis of data from multiple databases helped reduce the heterogeneity of individual datasets. The GSE76427 and TCGA-LIHC data downloaded from the GEO database were combined using the “merge” package (21). The “sva” package in R language was used to correct for differences and normalize for different sequencing batches (22), excluding patients with missing survival information. We finally obtained 371 patients with HCC from TCGA database and 115 patients from the GEO database, which were used for the subsequent analysis.

### 2.2 Clinical sample collection

We randomly collected 14 pairs of fresh HCC and adjacent normal tissue samples from the First Affiliated Hospital of Chongqing Medical University (Chongqing, China) between February and March 2023. In addition, 26 pairs of paraffin-embedded sections of HCC and para-cancerous tissues between June 2022 and December 2022 from the Pathological Diagnosis Center of Chongqing Medical University (Chongqing, China). None of the patients participating in our study underwent radiotherapy, chemotherapy, and immunotherapy before surgery. This study was approved by the Ethics Committee of the First Affiliated Hospital of Chongqing Medical University.

### 2.3 Cell culture and transfection

Human HepG2 and Huh7 HCC cells were purchased from the Cell Collection Center of the Chinese Academy of Sciences (Shanghai, China). All cells were maintained in Dulbecco’s Modified Eagle’s Medium (DMEM; Gibco, USA) containing 10% Fetal Bovine Serum (Wisent, Canada) and cultured at 37°C in a cell incubator with 5% CO2.

Lentiviruses targeting SLCO1B1 (forward, 5’-GGGGTACCATCATGGACCAAAATCAAC-3’, and reverse 5’-CTCGAGTGGAAACACAGAAGCAGAAG-3’) were purchased from GeneChem (Shanghai, China). Huh7 and HepG2 cells were transfected according to the manufacturer’s instructions. Stable strains were screened using 2 µg/ml puromycin. Three days after transfection, gene expression of the SLCO1B1 marker was observed under a fluorescence microscope, and cells with a transfection efficiency of >80% were selected for subsequent analysis.

### 2.4 Quantitative real time PCR

Total RNA was extracted from 14 pairs of fresh HCC and paraneoplastic tissues using the TRIZOL reagent (Takara Biotechnology Co., Ltd., Dalian, China) according to the manufacturer’s instructions. Total RNA was reverse transcribed into cDNA using the PrimeScrip™ RT kit (Takara Biotechnology Co., Ltd.). The polymerase chain reaction (PCR) was performed according to the manufacturer’s instructions. The amplification product was designed by Takara Biotechnology Co., Ltd. with the following sequence: SLCO1B1: forward, 5’-GAATGCCCAAGATGATGCTT-3’, and reverse, 5’-AATCCAGTGCAAGTGATTTCAAT-3’; β-actin: forward, 5’-AGAAAATCTGGCACCACACCT-3’, and reverse, 5’-GATAGCACAGCCTGGATAGCA-3’. Expression was normalized to that of β-actin and relative expression was calculated using the 2^-ΔΔCt^ method (23).

### 2.5 Immunohistochemistry stain

IHC was performed on 18 pairs of paraffin-embedded HCC and normal paracancerous tissue samples. The specific experiments were performed as previously described (24). Anti-human SLCO1B1 antibody (1:500, DF4534, Affinity Biosciences, China) was used to incubate the tissues overnight at 4°C. After application of the appropriate secondary antibody, the labeled antigen was visualized using a standard 3, 3’-diaminobenzidine (DAB) protocol. The slides were stained with hematoxylin. Two pathologists evaluated the staining results in a double-blind manner. The intensity of IHC staining was calculated from the intensity and number of stained cellular sections. The evaluation criteria for staining intensity were as follows: 0, 1, 2, and 3 represented negative, weak, moderate, and strong staining, respectively. The evaluation criteria for the number of stained cells were 0, 1, 2, 3, and 4, representing the percentages of stained cells as <10%, 10–25%, 25–50%, 50–75%, and >75%, respectively. IHC score = staining intensity × staining number. A score ≥6 is a high expression; otherwise, it is a low expression (25).

### 2.6 Multiplex immunofluorescence analysis

MIF detection of SLCO1B1 and CD86 was performed in pathological sections of HCC and adjacent normal tissues. First, the sections were deparaffinized and rehydrated, and antigen retrieval was performed using EDTA antigen retrieval buffer. Subsequently, the sections were incubated with 3% hydrogen peroxide at room temperature for 25 min in the dark to block endogenous peroxidase, and 3% Bovine Serum Albumin was used to block the sections for 30 min. Cyclic staining for both antigens in each section was then performed, including incubation with primary and secondary antibodies, fluorescence signal enhancement by Cyanine 3 Tyramide, and removal of Tyramide Signal Amplification (TSA) -antibody complexes using EDTA buffer. Subsequently, the cell nuclei were counterstained with DAPI for 10 min, and an autofluorescence quencher was added, reacted for 5 min, and rinsed with distilled water. Subsequently, sections were mounted in an anti-fluorescence quenching mounting medium. Finally, observed and collected images were obtained using a fluorescence microscope (Nikon ECLIPSE C1, Nikon DS-U3).

The scanned images were analyzed using the InForm software, and the results were independently analyzed by two experienced pathologists. The numbers 1, 2, and 3 represented low, medium, and high fluorescence intensities, respectively. The histochemical scoring formula was as follows: (high fluorescence intensity) × 3 + (median fluorescence intensity) × 2 + (low fluorescence intensity) × 1 (26).

We used SLCO1B1 and CD86 (Proteintech, 13395-1-ap) as primary antibodies, of which, SLCO1B1 was the key gene in our signature, and CD86 was the surface marker of M1 macrophages (27, 28). Goat Anti-Mouse IgG (H+L)-Alexa Fluor 488 was used as the secondary antibody (AIFang biological, SA002).

### 2.7 Colony formation assay

Cells (1×10^3^ cells per well in a six-well plate, and 5 ml of complete medium) was added to each well, shaken, mixed, and cultured in a cell incubator for approximately 14 days. The cells were fixed with 4% paraformaldehyde, stained with 1 ml of 0.5% crystal violet, rinsed with tap water, dried, and photographed.

### 2.8 Cell counting kit-8

First, we inoculated the well-growing cells into a 96-well plate, adjusted the cell concentration to 1.5×10 ^4^/ml, added 200 µl of cell suspension to each well, repeated three times for each group of cells, and place them in an incubator for culture. Then, after the cells adhered to the wall, 100 µl of CCK-8 working solution was added to each well, which was recorded as 0 h of the measurement, and the cells were placed in the incubator for 2.5 h. Finally, at specified time points (0, 24, 48, and 72 h), the absorbance value was measured at a wavelength of 450 nm by a microplate reader (Varioskan Flash, version: 4.00.53), and the cell viability curve was drawn according to the absorbance value.

### 2.9 Wound healing

First, we adjusted the cell concentration to 3×10 ^5^/ml, inoculated them into a 6-well plate, cultured in an incubator, observed that the cell confluence reached 100%, took a 200 µl sterile pipette tip and drew three vertical lines, and washed the exfoliated cells using the sterile phosphate buffered saline, and then, 2 ml DMEM was added to each well. Finally, the scratches were observed under a microscope and photographed at 0, 12, and 24 h.

### 2.10 Differential analysis, genomic characteristics of DRGGs and drug sensitivity analysis of DRGGs subtypes

We retrieved 14 DRGs from the relevant literature (**Supplementary Table 1**), 326 GRGs were extracted from the MSigDB website (https://www.gsea-msigdb.org/gsea/msigdb/) (**Supplementary Table 2**). Then, we normalized the data from TCGA and GEO databases using the “limma” and “survival” package of R language, and obtained 45 disulfidptosis-related glycolytic genes (DRGGs) with the screening condition of |cor|>0.65 (29). Then, the frequency and type of mutations of 45 DRGGs in patients with HCC were analyzed by the “maftools” package, and the results were presented as “waterfall plots” (30). In addition, the somatic copy number variation (CNV) frequencies of the above genes were shown by “bubble plots”, and the sites where the mutations occurred were shown by “circle plots”. Next, in order to clarify the sensitivity of patients with DRGGs molecular subtypes of HCC to chemotherapy drugs, we calculated the drug concentration values when half of the cells were induced to undergo apoptosis by drugs for the treatment of HCC (IC50) using the “pRRophetic” package (31).

### 2.11 Consensus clustering analysis of DRGGs

Consensus clustering analysis was performed using the “ConsensusClusterPlus” R language package to classify the enrolled patients with HCC were divided into different molecular subtypes according to the differential expression of DRGGs (32). Intragroup associations were enhanced and intergroup associations were reduced after clustering. Subsequently, heterogeneity between the two groups was described by principal components analysis (PCA) and cumulative density functional (CDF) curves. To assess the value of consistent clustering analysis in the treatment of patients with HCC, we compared the between-group differences in clinicopathological characteristics of patients with different subtypes by heat map. Kaplan-Meier (K-M) curves were used to determine survival differences between the two subtypes by the “survival” and “survminer” package in R Studio. To clarify the functional differences between the two subtypes, gene set variation analysis (GSVA) was performed by the Kyoto Encyclopedia of Genes and Genomes (KEGG). In addition, differences in immune cell infiltration were analyzed using single-sample gene set enrichment analysis (ssGSEA) to understand the differences in the immune microenvironment between the groups.

### 2.12 Screening, functional analysis and prognostic analysis of differential genes between molecular subtypes of DRGGs

Differentially expressed genes (DEGs) between molecular subtypes of DRGGs were screened using the “limma” package in R language with FDR<0.05 and |log2fold change (FC)|≥0.585 as criteria. Functional enrichment analysis was conducted using Gene Ontology (GO) and KEGG to further explore the potential gene functions and enrichment pathways of DRGGs.

Next, differential genes with prognostic value between the two subtypes were screened by univariate Cox regression analysis, and patients were classified into different genetic subtypes based on these genes. Survival analysis was performed using K-M to verify the prognostic differences between different gene subtypes. In addition, differences in clinicopathological characteristics between patients with different subtypes were assessed to guide the direction for targeted therapy.

### 2.13 Construction of a prognostic signature

First, genes with prognostic value were screened using univariate Cox regression analysis, and the accuracy of the signature was improved using LASSO regression analysis. Independent prognostic factors associated with HCC were screened based on multivariate Cox regression analysis, and the risk score was calculated using the multivariate Cox regression coefficients and the expression of DRGGs in patients with HCC. Then, the prognostic signature was constructed. The scoring formula was as follows:


risk score =∑(Expi ∗ coefi)

where Expi and coefi represent the expression of genes and regression coefficients, respectively. Subsequently, all patients with HCC were randomly divided into a training group and test group at a 1:1 ratio. Then, the patients were further classified into high-risk and low-risk groups based on their median of the prognostic scores.

### 2.14 Analysis and validation of clinical relevance of the prognostic signature

First, we calculated the differences in risk scores across the DRGGs molecular subtypes and gene clusters to assess whether the risk score retained its predictive power across subgroups. Differential expression maps of DRGGs between the high- and low-risk groups were constructed using the “ggplot2” package. The prognostic value of clinicopathological elements and risk scores was assessed by Cox regression analysis. Next, survival differences between patients in various risk groups were identified using the K-M survival analysis, plotting receiver operating characteristic (ROC) curves to assess the diagnostic value of the scoring system. Then, the accuracy of the results was further validated in the test group.

### 2.15 Creation and verification of nomogram

To evaluate the prognostic characteristics of patients at 1-, 3- and 5-year, the “rms” and the “regplot” packages of R language were used to construct the nomogram by combining clinical features such as age, gender, risk scores and tumor stage of patients. Each patient’s clinical information corresponded to a score and the total score was the sum of each index used for the scoring system of the nomogram. Finally, the scores were used to assess the probability of survival at 1-,3-,5-year intervals.

### 2.16 Exploration of tumor immune microenvironment

The main characteristics of the TIME include the extent of immune cells infiltrate, expression profile of immune checkpoints, and activity of anti-cancer immune responses. First, we assessed the relation between risk scores and the proportion of immune cells infiltration in patients with HCC using Spearman’s correlation analysis. We also used the “CIBERSORT” package in R language to quantify the enrichment of different immune cells in each tumor sample and analyzed the relationship between genes and immune cells in the signature. To further understand the differences in the TIME between different risk groups and their relevance to immunotherapy, we evaluated the differences in immune checkpoint expression between the high- and low-risk groups. In addition, the ESTIMATE algorithm was applied to calculate the stromal, immune and estimated scores in the two risk groups, reflecting the degree of stromal and immune cell infiltration and tumor purity for each risk group, respectively, and a violin plot was used to visualize the differences between groups. Besides, we evaluated the enrichment of immune-related pathways in the different groups using gene set enrichment analysis (GSEA) and the activity of the seven steps of the anticancer immune response using ssGSEA to understand the role of risk scores in the TIME and thus assess tumor prognosis (33, 34).

### 2.17 Exploration of genomic features in prognostic signature

We applied mutation data downloaded from TCGA-LIHC to analyze the tumor mutation burden (TMB) and major mutation types in the different risk groups. TMB has emerged as a biomarker to forecast the efficacy of immunotherapy (35). In addition, it has been shown that microsatellite instability (MSI) is associated with tumorigenesis, generally caused by DNA replication defects (36). We used MSI analysis between different risk groups as a reference for prognostic assessment. The poor prognostic of HCC is intimately associated with the emergence of drug resistance, and researches on cancer stem cells (CSCs) indicate that tumor development was driven by a fraction of stem cells; therefore, it is crucial to explore the stemness of CSCs (37). We assessed the degree of resemblance between stem cells and tumor cells by calculating mRNAsi to quantify the association between CSCs and risk scores.

### 2.18 Statistical analysis

We analyzed the data using the R language software (version 4.2.2), performed t-tests for normally distributed data, and applied Spearman’s test for correlation analysis. GraphPad Prism software (version 8.0.1) was used for plotting the images, with P<0.05 as the threshold of significance for all statistical analyses.

## 3 Results

### 3.1 Characterization and expression of DRGGs mutations in HCC

**Figure 2:**
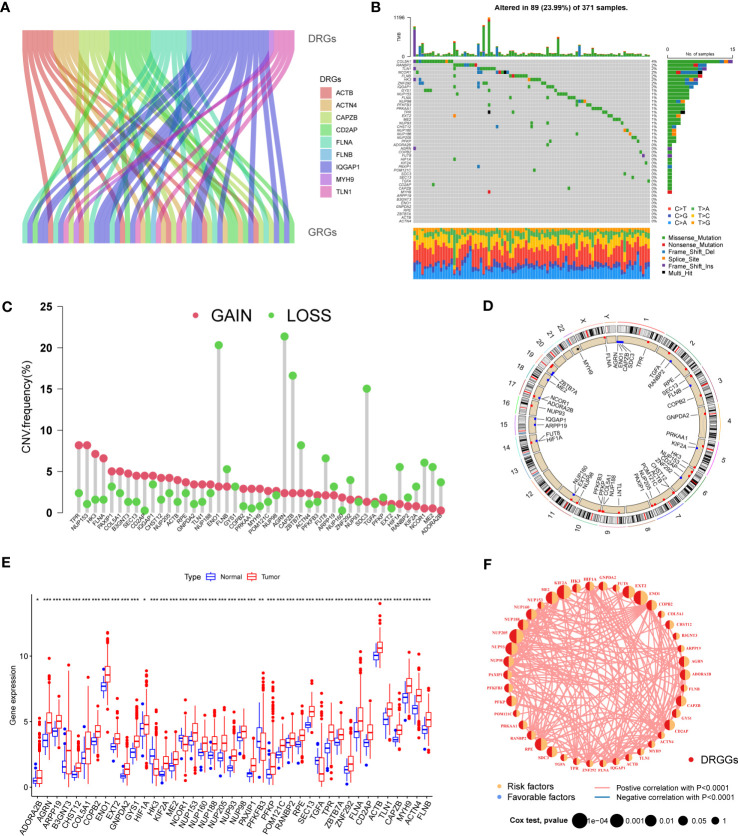
**(A)** The Sankey diagram showing the correlation between DRGs and GRGs. **(B)** Mutation frequencies and mutation types of 45 DRGGs in 371 patients with HCC from the TCGA database. **(C)** Frequency of increased and decreased CNV in DRGGs. **(D)** Location of CNV of DRGGs on 24 chromosomes. Red dots indicate increased copy number and blue dots indicate decreased copy number. **(E)** Expression of 45 DRGGs between normal and HCC tissues. * represents P<0.05, ** represents P<0.01, *** represents P<0.001. **(F)** Interaction relationship between DRGGs in HCC. The thickness of the connecting line indicates the strength of the correlation effect between genes, and the pink color represents positive correlation.

First, we demonstrated the interactions between DRGs and GRGs using a Sankey diagram (**Figure 2A**). TMB analysis of DRGGs showed that 89 (23.99%) of the 371 patients had mutations. Among these, the COL5A1 mutation frequency was the highest (4%), followed by RANBP2 (**Figure 2B**). Next, the somatic CNV frequency of DRGGs in HCC was further evaluated and copy number alterations were found in all gene numbers. Among them, most genes, such as TPR, NUP153, HK3, FLNA and PAXIP1, had increased CNV frequencies, whereas ENO1, FLNB, AGRN, CAPZB and ZBTB7A had decreased CNV (**Figure 2C**). In addition, we showed the location of CNV of DRGGs occurring on chromatin by a ring plot (**Figure 2D**) and found that most DRGGs were located on chromosomes 1, 2, 3, and 7. Besides, we compared the expression of DRGGs between HCC tissues and normal samples and found that most genes, such as AGRN, B3GNT3 and FLNA, were highly expressed in tumor tissues (**Figure 2E**), resulting in a worse patient prognosis (**Figures S1A–P**, **Figures S2A–P**, **Figures S3A–E**). **Figure 2F** shows that DRGGs are positively correlated and play a promoting role in HCC progression. In addition, most genes were positive associated with CNV changes, indicating that CNV may be one of the factors affecting gene expression levels (**Figures 2E, F**). Thus, the analysis of mutations and expression of DRGGs showed significant differences between HCC and normal tissues, indicating that this gene cluster may play a key role in HCC progression.

### 3.2 Construction and prognostic analysis of molecular subtypes of DRGGs in patients with HCC

**Figure 3:**
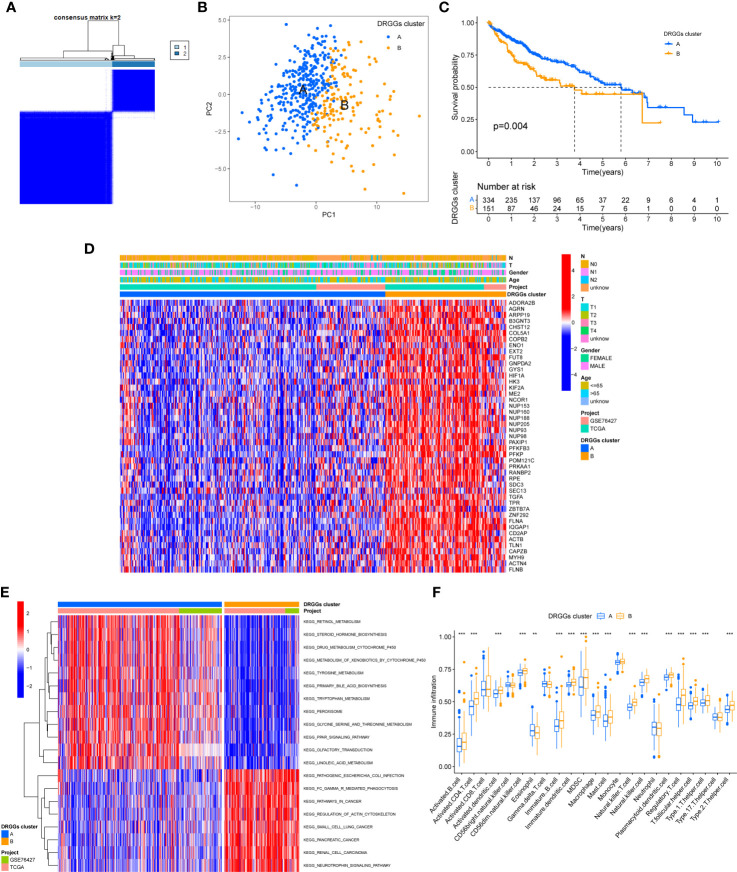
**(A)** Diagram of the consensus matrix defining the correlated regions of the two subtypes. **(B)** PCA analysis showing significant differences between the two subtypes. **(C)** K-M analysis showing the prognostic characteristics of patients in both subtypes. **(D)** Differences in clinicopathological features and expression levels of DRGGs between the two different subtypes. **(E)** GSVA of biological pathways between the two different subtypes, red and blue represent activating and inhibiting pathways, respectively. **(F)** The extent of infiltration of 23 immune cells in HCC subtypes. PCA, principal component analysis; GSVA, gene set variation analysis; K-M, Kaplan-Meier. ** represents P<0.01, *** represents P<0.001.

We evaluated the HCC subtypes based on differences in the expression of DRGGs and performed a cluster analysis of patients with HCC using TCGA-LIHC and GEO (GSE76427) databases. During the cluster analysis of the 486 samples, k=2 was considered the best clustering method to minimize the differences between groups, and the patients with HCC were divided into two subtypes: DRGGs cluster A and DRGGs cluster B (**Figure 3A**). Besides, the results were verified by PCA (**Figure 3B**) and CDF curves (**Figure S3F**). In addition, the tracking plot showed that the sample was the most stable when k = 2 (**Figure S3G**). In the K-M survival analysis of patients with both subtypes, it was found that the DRGGs cluster A had a better survival outcome (**Figure 3C**).

### 3.3 Gene set variation analysis and TIME analysis of molecular subtypes of DRGGs

First, we plotted a heat map using clinicopathological information, which showed the relationship between sex, age, T and N stages, and DRGGs cluster, where DRGGs were highly expressed in DRGGs cluster B and almost all of them were oncogenes (**Figure 3D**), explaining the adverse prognosis of DRGGs cluster B patients. Then, GSVA analysis of the two subtypes was performed using KEGG to compare the variation in the enrichment pathways, it was found that DRGGs cluster A was highly enriched in the drug metabolism cytochrome P450, steroid hormone biosynthesis, tyrosine metabolism, PPAR signaling pathway, whereas the remaining pathways, such as cancer pathway, pathogenic E. coli infection and actin cytoskeleton regulation were highly enriched in DRGGs cluster B. (**Figure 3E**). Besides, we explored the variation in the degree of the immune cell infiltration for both subtypes by ssGSEA. Most of the 23 immune cells were highly infiltrated in DRGGs cluster B (**Figure 3F**). However, patients with cluster B had a significantly lower CD8 T cell/T cell regulatory (Treg) ratio than patients with subtype A, resulting in a poorer prognosis (**Figure S3H**).

### 3.4 Drug sensitivity analysis

**Figure 4:**
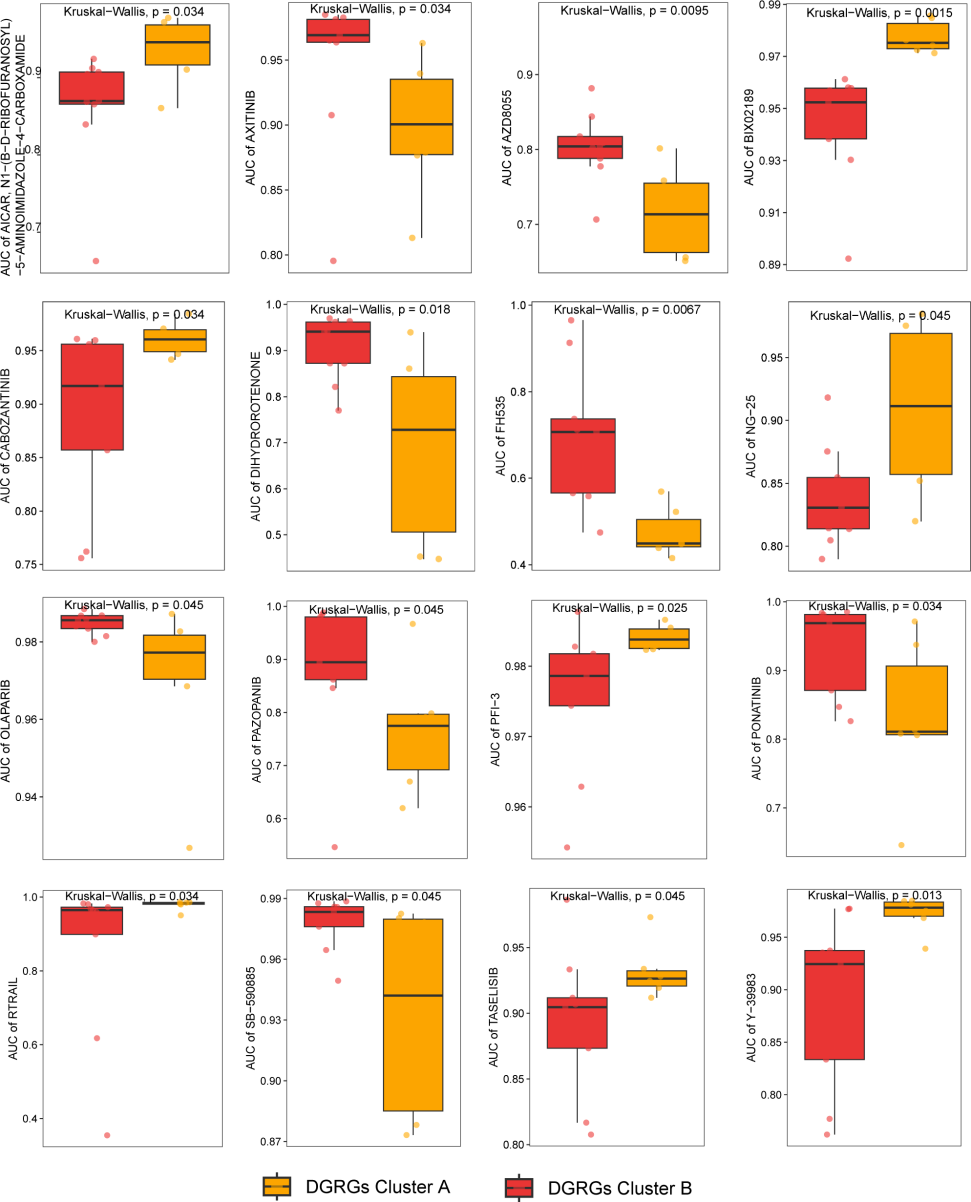
The relationship between patients with different DRGGs subtypes and chemotherapy sensitivity.

We explored the sensitivity of patients with HCC with the two DRGGs molecular subtypes to the chemotherapy drugs commonly used to treat HCC, and found that patients with DRGGs cluster A were sensitive to AICAR, BIX02189, CABOZANTINIB, NG-25, PFI-3, RTRAIL, TASELISIB, and Y-39983 drugs. However, patients in DRGGs cluster B were more sensitive to AXITINIB, AZD8055, DIHYDROROTENONE, FH535, OLAPARIB, PAZOPANIB, PONATINIB, and SB−590885 (**Figure 4**). There were significant differences in drug sensitivity among different subtypes of HCC, which could provide direction for personalized treatment of HCC.

### 3.5 Construction of gene subtypes based on differential genes between molecular subtypes of DRGGs and validation

**Figure 5:**
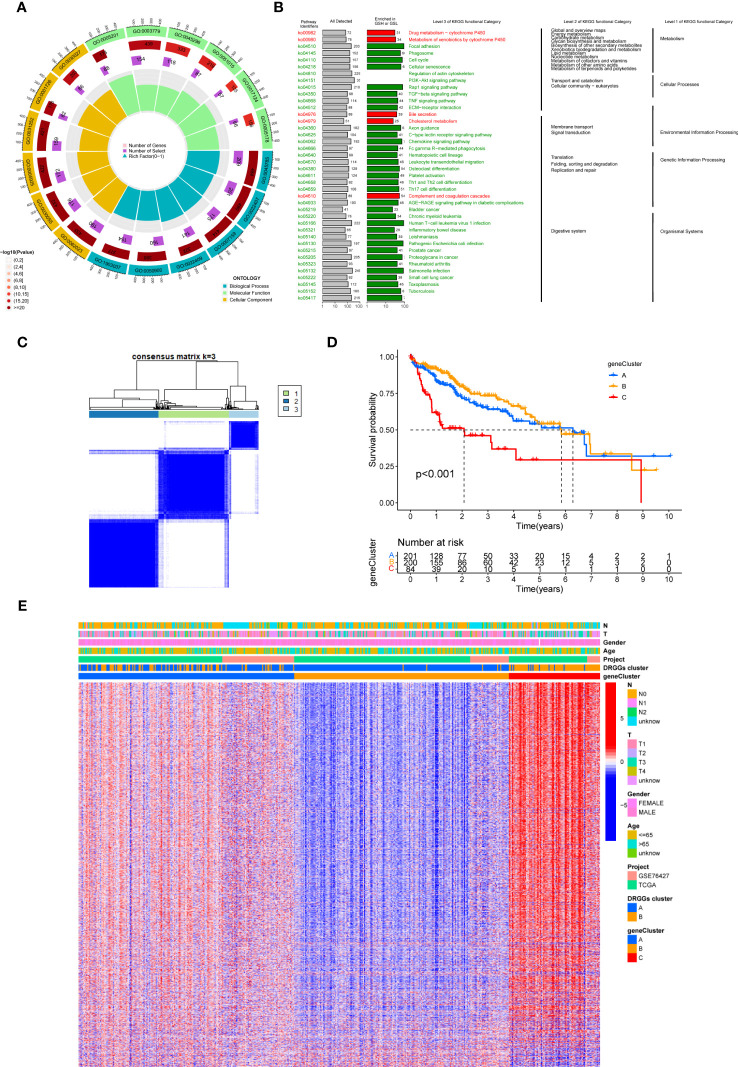
**(A)** GO enrichment analysis of DEGs between two DRGGs subtypes. The red part of the graph represents the number of enriched genes and the redder the color, the more significant gene enrichment; the purple part represents the number of enriched differential genes. The bar graph represents the proportion of genes. **(B)** KEGG enrichment analysis of DEGs between two DRGGs subtypes. **(C)** Diagram of the consensus matrix defining the correlated regions of three cluster-related regions. **(D)** K-M curves for the three gene subtypes. **(E)** Relationship between the three gene subtypes and clinicopathological features.

First, to detect the possible biological behavior of tumor cells, we screened a total of 3451 differential genes between DRGGs cluster A and cluster B by “BiocManager” and “limma” packages in R Studio. Next, using GO functional enrichment analysis, we found that the differential genes were mainly enriched in biological processes (BP) functional set, such as cytoplasmic translation and xenobiotic metabolic processes, and associated with cellular component (CC), such as cell-substrate junction and focal adhesion. As for molecular function (MF), extracellular matrix structural constituent and actin binding played an essential part in neoplasm proliferation (**Figure 5A**) (**Supplementary Table 3**). Next, The KEGG enrichment analysis was conducted on the different genes, and the findings showed that the main pathways were focused on metabolism, membrane transport, signal transduction, genetic information processing, and other related pathways (**Figure 5B**) (**Supplementary Table 4**). Therefore, DRGGs play an essential role in HCC progression.

**Figure 6:**
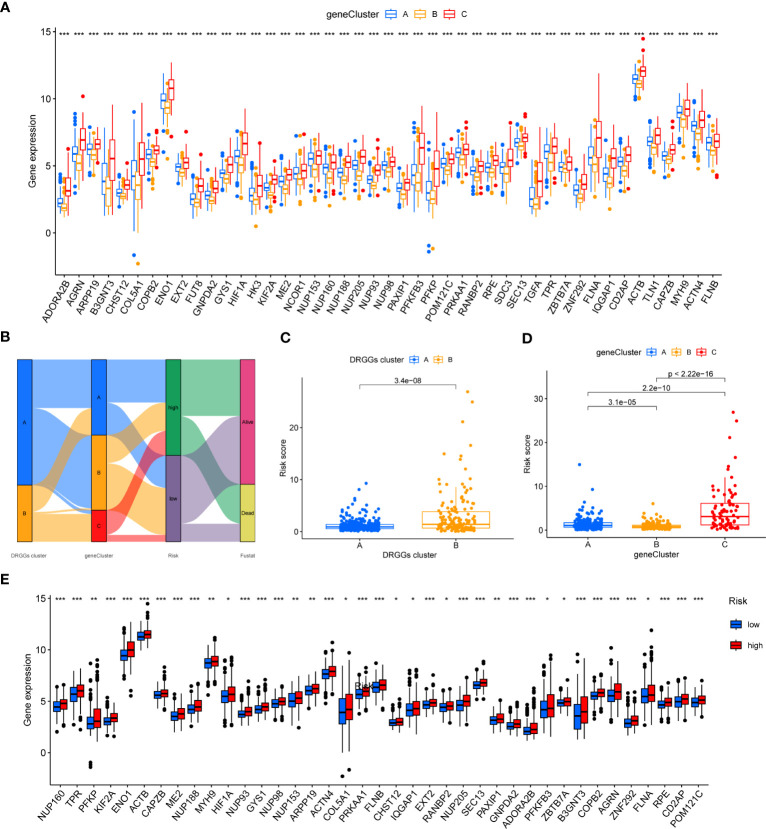
**(A)** Differential expression of 45 DRGGs in the three gene subtypes. **(B)** Sankey diagram of different HCC subtypes and survival outcomes. **(C)** Differences in risk score among DRGGs subtypes. **(D)** Risk score differences among different gene subtypes. **(E)** Expression differences of 45 DRGGs in high-risk and low-risk groups. * represents P<0.05, ** represents P<0.01, *** represents P<0.001.

We then acquired 1,167 genes with prognostic value using univariate Cox regression analysis. To further validate this regulatory mechanism, the samples were typed again according to the 1167 prognostic genes, and the clustering diagram was obtained using the “ConsensusClusterPlus” algorithm in R language. K=3 was the best clustering method for the samples (**Figure 5C**), and three genetic subtypes were obtained, namely gene clusters A, B and C. The CDF curves verified the clustering accuracy (**Figure S3I**). K-M analysis suggested that patients with gene cluster C had the worst prognosis, whereas those with gene cluster B had a higher survival rate (p < 0.001) (**Figure 5D**). A heat map of the clinicopathological features showed that gene cluster C mainly corresponded to DRGGs cluster B, and that patients with both subtypes had the worst prognosis (**Figure 5E**). Additionally, analysis of the expression of DRGGs in patients with the three gene subtypes revealed that the expression of DRGGs decreased sequentially in gene cluster C, gene cluster A, and gene cluster B, with statistically significant differences (P<0.001) (**Figure 6A**).

### 3.6 Construction and validation of risk prognostic signature

First, we constructed a prognostic signature for DRGGs from the differential genes among the three gene subtypes based on significant gene data obtained from multifactorial Cox regression analysis using LASSO regression analysis to avoid overfitting (**Figures S3J, K**). Seven genes included were ETV5, FZD7, CD5, SLCO1B1, CD79A, SNX7, and SLC1A7, and the risk score equation was: Risk score = (0.3039 * expression of ETV5) + (0.3091 * expression of FZD7) + (-0.2449 * expression of CD5) + (-0.1656 * expression of SLCO1B1) + (-0.3676 * expression of CD79A) + (0.2673 * expression of SNX7) + (0.1420 * expression of SLC1A7). Besides, Sankey plots indicated a consistent relationship among the two molecular subtypes of DRGGs, the three genetic subtypes, the different risk groups for prognostic features, and the prognosis of patients (**Figure 6B**). Next, we assessed the association between the three gene subtypes and risk scores and observed that gene cluster B had the lowest risk score, whereas cluster C had the highest risk score. More importantly, DRGGs cluster B exhibited a higher risk score compared to DRGGs cluster A, consistent with data from previous survival analyses (**Figures 6C, D**). In addition, DRGGs were high expression in the high-risk group, further confirming the accuracy of the differences between HCC and normal adjacent tissues (**Figure 6E**).

**Figure 7:**
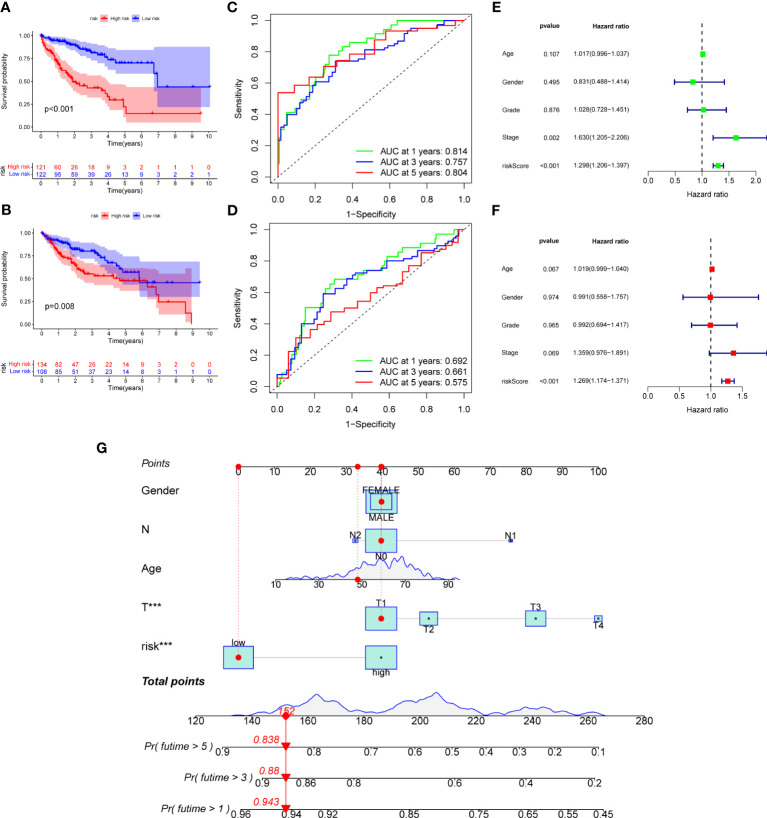
**(A)** K-M analysis of the Recurrence free survival (RFS) of high-risk and low-risk patients in the train group. **(B)** K-M analysis of the RFS of high-risk and low-risk patients in the train group. **(C)** ROC curves for predicting the 1-, 3-, and 5-year survival rates of patients in the train group. **(D)** ROC curves for predicting the 1-, 3-, and 5-year survival rates of patients in the test group. **(E)** The univariate Cox regression analysis of clinical characteristics and risk score in the train group. **(F)** The multivariate Cox regression analysis of clinical characteristics and risk score in the train group. **(G)** Construction of a nomogram based on clinical characteristics and risk score for prognostic signature. RFS, recurrence free survival; ROC, receiver operating characteristic.

Next, we further validated the value of the prognostic signature. The results of survival analysis revealed remarkably shorter survival times in the high-risk group, both in the overall study cohort and in the train and test groups (P<0.01) (**Figures 7A, B**, **Figure S3L**). ROC analysis of all patients with HCC according to the prognostic signature showed that the areas under the curve (AUC) were 0.753, 0.708 and 0.666 at 1-, 3- and 5- years, respectively (**Figure S3M**). In the train group, the 1-, 3-, and 5-year AUCs were 0.814, 0.757, and 0.804, respectively (**Figure 7C**), whereas those in the test group were 0.692, 0.661, and 0.575, respectively, strongly confirming the diagnostic power of the signature (**Figure 7D**). Subsequently, we found the prognostic value of the tumor stage and risk score in the train group using univariate Cox regression analysis. Multivariate Cox regression analysis suggested that the risk score was an independent prognostic factor in all groups (**Figures 7E, F**).

### 3.7 Creation of nomogram

Owing to the limitations of the scoring system alone in clinical application, we integrated risk scores with the clinical information of patients to create a nomogram to predict patients’ survival time at 1-, 3- and 5- years. Both T-stage and risk scores were independent prognostic factors (**Figure 7G**). A calibration chart further confirmed the accuracy of the signature (**Figure S3N**).

### 3.8 Assessment of TIME and biological characteristics between the risk groups

**Figure 8:**
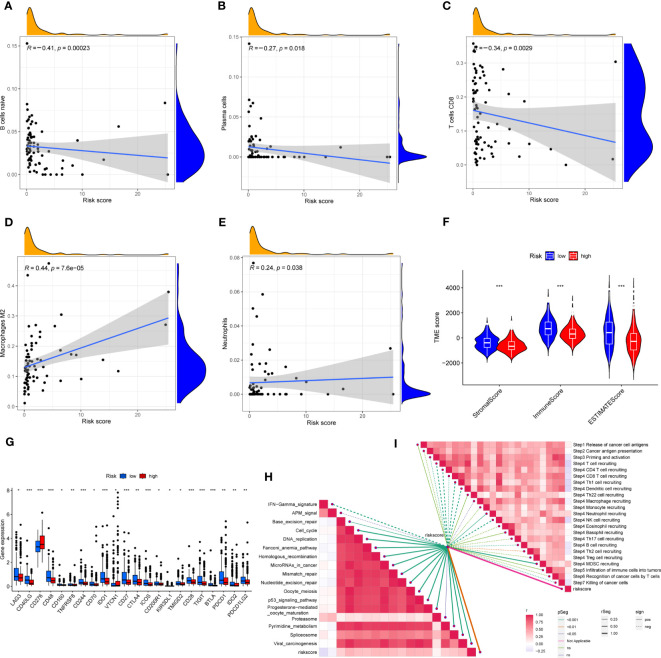
**(A–E)** Correlation of risk score with immune cells. **(F)** Correlation of risk score with immune score, stromal score and tumor purity. **(G)** Differences in the expression of immune checkpoints in the high-risk and low-risk groups. **(H)** The correlation between risk score and the enrichment of the relevant pathways for immunotherapy. **(I)** The correlation between risk score and the steps of the cancer immunity cycle. * represents P<0.05, ** represents P<0.01, *** represents P<0.001.

First, correlation between the risk scores and immune cells was visualized using scatter plot. The results showed that naive B cells, CD8+ T cells and plasma cells negatively related to risk scores, whereas M2 macrophages and neutrophils positively associated with risk scores (**Figures 8A–E**). In addition, the TME scores indicated that the low-risk group had higher tumor purity, stromal and immune scores (**Figure 8F**). Second, the differences in immune checkpoint expression suggested that most immune checkpoint molecules such as CD40LG, CD48, IDO1, CD27, PDCD1 were strongly expressed in the low-risk population. However, CD276 was expressed highly in the high-risk population (**Figure 8G**), suggesting that immune checkpoints were involved in tumor progression and are promising applications in the low-risk population to help guide immunotherapy. Finally, based on the study by Jiao Hu et al., we obtained the steps of the cancer immunity cycle and the enrichment scores of the immunotherapy-predicted pathways dataset (38). The “ggcor” package was used to construct the correlation of the risk scores with the dataset. The results showed that the IFN-γ signaling pathway was mainly concentrated in the low-risk group, and p53 signaling pathway, cell cycle, DNA replication, and microRNAs in cancer were more significantly enriched in the high-risk group (**Figure 8H**) (**Supplementary Table 5**). In addition, the risk score was mostly negatively correlated with the steps of the cancer immunity cycle, including the recruitment process of T cell, CD4+ T cell, Th1 cell, dendritic cell, and NK cell, whereas the recruitment of neutrophil was more active in the high-risk group (**Figure 8I**) (**Supplementary Table 6**).

### 3.9 Relationship between risk scores and TMB, MSI, and CSCs

**Figure 9:**
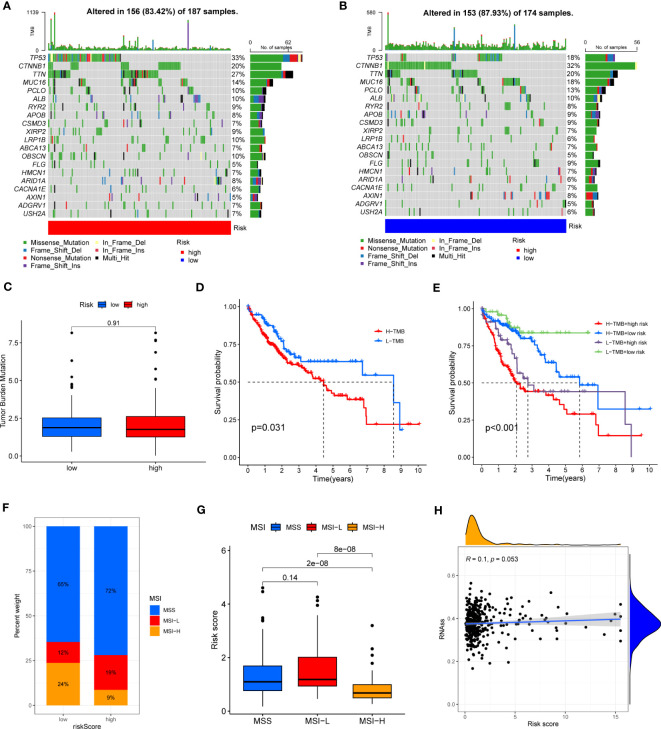
**(A, B)** Waterfall plots of somatic mutation frequency and mutation type between different risk groups. Each column represents an individual patient. The bar above each column shows the TMB, numbers on the right side indicates the mutation frequency of each gene, and the bars on the right show the proportion of each mutation type. **(C)** TMB differences in different risk groups. **(D)** Differences in survival between the high TMB and low TMB groups. **(E)** Survival differences between patients assessed by TMB and risk score combined. **(F, G)** Relationship between risk score, MSS and MSI. **(H)** Relationship between risk score and CSCs.

HCC development is influenced by multiple complex factors, including TMB, MSI, and CSCs. Therefore, it is crucial to explore the relationships between the prognostic signature and these factors. It has been suggested that patients with higher TMB may have stronger immunogenicity and thus higher sensitivity to immunotherapy (39). Therefore, we included 361 HCC patients with complete mutation information from the TCGA database, counted the number of variants and mutation types in each sample. The top 20 genes in terms of mutation frequencies were selected using waterfall plots. Comparative analysis of different risk groups showed that TMB occurred in 87.93% of patients in low-risk group, with the most significant mutation in CTNNB1 (32%). However, TMB occurred in 83.42% of the patients with HCC in the high-risk group, with the most significant mutation in TP53 (33%). Besides, the difference in TMB between the two risk groups was not statistically significant (P=0.91) (**Figures 9A–C**). However, it is worth mentioning that survival analysis suggested a better prognosis in the low TMB group (P<0.05) (**Figure 9D**). In addition, by combining the risk score and the TMB from the prognostic signature, survival analysis showed statistically significant survival among the four groups (p<0.001) (**Figure 9E**). In conclusion, there was no significant difference in TMB between the high-risk and low-risk groups, but TMB combined with risk score was a better predictor of overall survival time.

In addition, it has been shown that for oncology patients, the higher the MSI, the higher the potential for selecting immunotherapy (40). It has been suggested that MSI is a biomarker for determining response to immune checkpoint therapy (41). Our analysis of patients with HCC showed that the MSI-H group had a lower risk score than the MSS and MSI-L groups (P<0.001) (**Figures 9F, G**).

Besides, we assessed the association between CSCs and the signature risk score. The results showed no statistically significant relationship (r = 0.1, p = 0.053). These suggested that the differential tumor stemness of patients between the two risk groups was not significant and that the prognosis of patients with HCC was mainly influenced by a combination of other factors (**Figure 9H**).

### 3.10 Expression and immune infiltration characteristics of 7 genes in the signature

**Figure 10:**
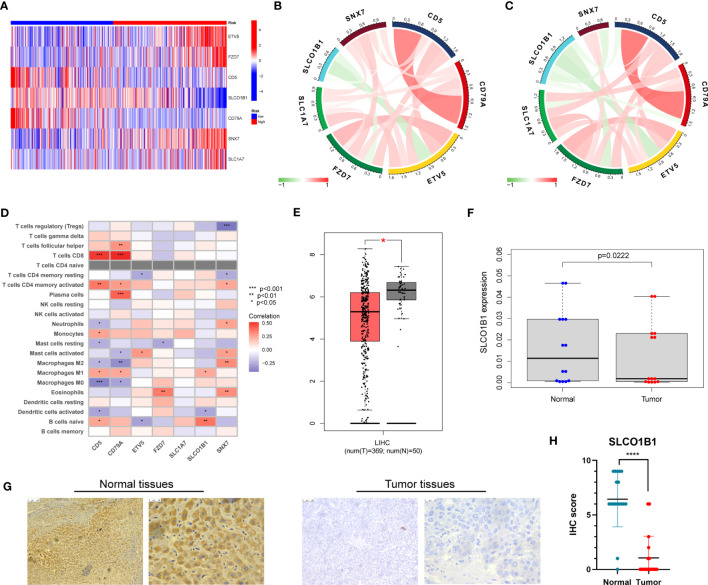
**(A)** Expression of the seven genes in the risk signature in the high-risk and low-risk groups. **(B)** Correlation of the seven genes in the train group. **(C)** Correlation of the seven genes in the test group. **(D)** Correlation between the level of immune cell infiltration and the seven genes in the risk model. **(E)** Differential expression of SLCO1B1 in HCC tissues and normal tissues. The red part represents HCC patient samples, and the gray part represents normal patient tissue samples. **(F)** The result of qRT-PCR. **(G)** Normal and cancer images of SLCO1B1 expression in liver tissues (100× and 400×) detected by IHC staining. **(H)** IHC score for all samples. **** represents P<0.0001.

First, we evaluated the differential expression of the seven genes in different risk groups, and the results are shown in **Figure 10A**. We then explored the association of the seven genes in the training group and discovered that SLCO1B1 was negatively correlated with the other six genes and positively correlated with the remaining six genes (**Figure 10B**), in accordance with the validation results of the test group (**Figure 10C**). In addition, we evaluated the relationship between seven genes in the signature and immune cells and found that CD5, CD79A, SNX7, and SLCO1B1 were relatively strongly correlated with immune cells, especially CD5, CD79A, and SNX7 (**Figure 10D**).

**Figure 11:**
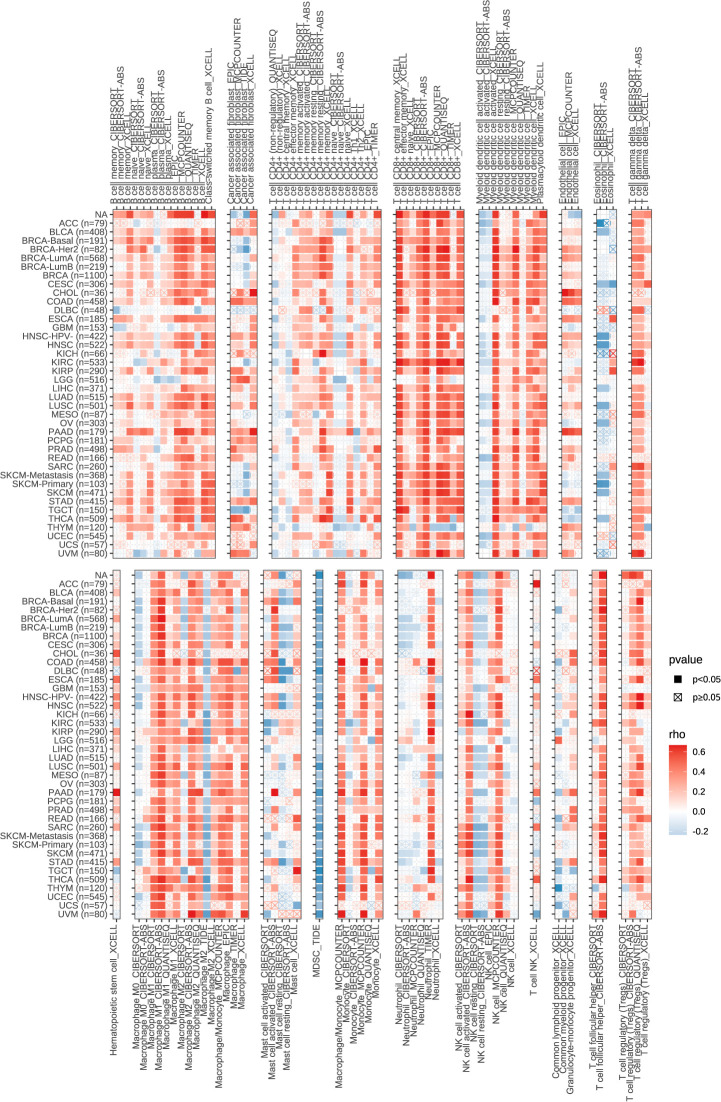
Correlation of SLCO1B1 expression with the level of infiltration of various immune cells in cancers.

**Figure 12:**
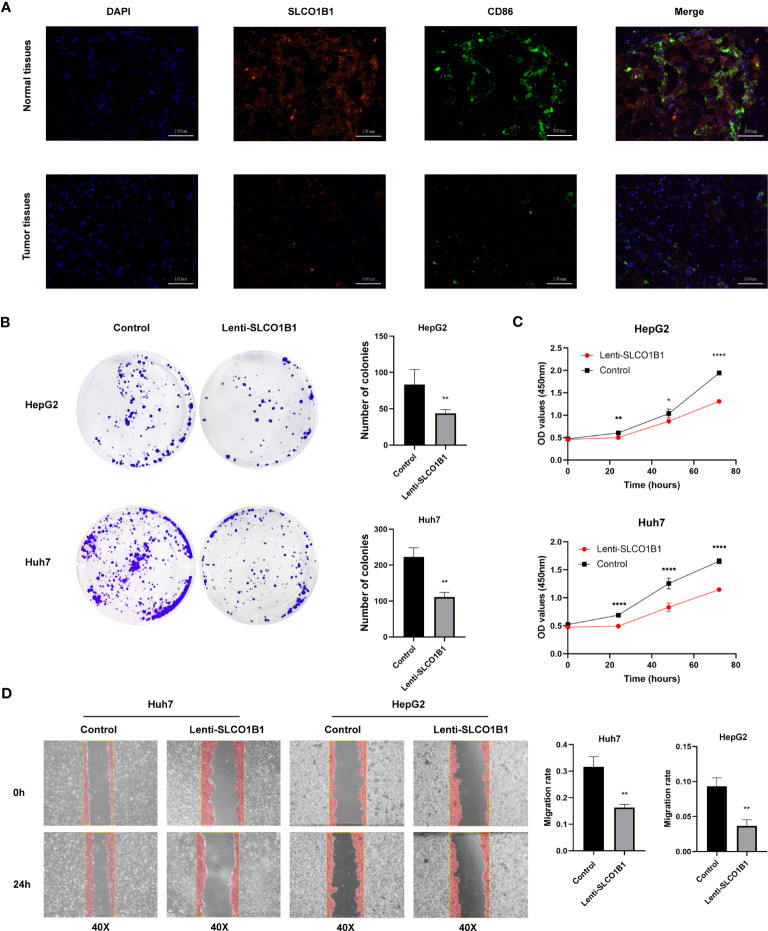
**(A)** Representative immunofluorescence images (magnification: ×200) of the SLCO1B1 and CD86 expressions in HCC and normal adjacent tissues. DAPI, 4′,6-diamidino-2-phenylindole. **(B)** Colony formation of control group and Lenti-SLCO1B1 group. **(C)** The viability of HCC cells at 0 h, 24 h, 48 h, 72 h was detected by Cell counting kit-8. (*p < 0.5, **p < 0.01, ****P < 0.0001). **(D)** Wound Healing of control group and Lenti-SLCO1B1 group.

Next, we explored the expression of these seven genes in HCC samples and paracancerous tissues using the data and found that SLCO1B1 was expressed at low levels in HCC samples (P<0.05) (**Figure 10E**), and the differential expression of the remaining genes was not statistically significant. Therefore, we verified the expression levels of SLCO1B1 based on qRT-PCR and IHC, which showed low expression in HCC samples (**Figures 10F–H**) (**Supplementary Table 7**), and the results were in accordance with the data from TCGA. In addition, through pan-cancer analysis, we further demonstrated that SLCO1B1 has a high immune infiltration status in most tumors, especially in B cells, dendritic cells, CD8+ T cells, macrophages, Tregs, and T-cell follicular helper cells. In addition, M1 macrophages, NK cells, and CD8+ T cells showed significant infiltration into the HCC (**Figure 11**). The above results indicate that SLCO1B1 is strongly correlated with M1 macrophages, and inducing the polarization of the TIME to the tumor-suppressive M1 phenotype is the key to improving the effect of immunotherapy (42, 43). Therefore, we selected the surface marker CD86 of M1 macrophages and evaluated the localization and expression of CD86 and SLCO1B1 in the liver tissue by mIF. The results showed that the expression levels of CD86 and SLCO1B1 in HCC tissues were downregulated and were positively correlated (**Figure 12A**).

### 3.11 SLCO1B1 inhibits the proliferation, migration and invasion of HCC cells *in vitro*

We confirmed low expression of SLCO1B1 in HCC tissues. To further explore the biological function of SLCO1B1, we first constructed HCC cell lines overexpressing SLCO1B1 and then conducted a series of experiments to explore whether SLCO1B1 could regulate tumor cell proliferation and migration. The results of the colony formation experiments showed that overexpression of SLCO1B1 inhibited colony formation in HepG2 and Huh7 cells compared to the control group (**Figure 12B**). In addition, the CCK-8 assay showed that the overexpression of SLCO1B1 inhibited the proliferation of HepG2 and Huh7 cells (**Figure 12C**). In addition, the results of the migration experiments showed that overexpression of SLCO1B1 inhibited the migration ability of HCC cells (**Figure 12D**). Collectively, these results suggested that SLCO1B1 inhibited the proliferation, migration, and invasion of HCC cells.

## 4 Discussion

HCC is a highly heterogeneous tumor, with considerable variation in genomics, transcriptomics, proteomics, and metabolomics (44). Disulfidptosis is a recently identified pattern of programmed cell death in which excessive accumulation of intracellular cystine leads to disulfidptosis. Tumor cells expedite the reduction of ingested cystine to cysteine to avoid disulfidptosis (45). Several studies have demonstrated the potential of targeting disulfidptosis in tumor therapy (6, 8). Additionally, the immunomodulatory drug dimethyl fumarate (DMF) targets glycolysis by catalyzing cysteine, which acts as an anti-inflammatory agent (46). The glycolytic enzyme GAPDH is also involved in regulating the glycolytic process by catalyzing cysteine production during the tricarboxylic acid cycle, and many GRGs have been identified as effective prognostic markers of HCC (47, 48). However, the roles of genes related to disulfidptosis and glycolysis in HCC has not been well-studied.

In this study, we explored the correlation between DRGGs and HCC. Surprisingly, these genes were not significantly mutated in HCC. However, their differential expression between HCC and normal tissues is equally important. Subsequently, HCC patients were divided into two distinct molecular subtypes based on DRGG expression. The pathological staging and overall survival time of patients with DRGG cluster B were not satisfactory compared to those of patients with cluster A. In addition, there were significant differences in gene expression, pathway enrichment, and immune cell infiltration between the two subtypes. In particular, patients with cluster B had lower CD8+ T cell/Treg ratios, leading to a poorer prognosis, as demonstrated in a previous HCC study (49). In addition, by constructing a risk-prognosis signature we found that patients in the high- and low-risk groups had significant differences in clinicopathological characteristics and prognosis by constructing a risk prognosis signature, which would help clinicians evaluate the prognostic characteristics of patients and formulate targeted treatment plans.

Several factors influence the expression of prognosis-related genes in HCC. Among the seven genes used to construct the signature, SLCO1B1 was expressed at low levels in HCC tissues, whereas the expression of the remaining genes did not differ significantly between HCC and normal tissues. However, from a prognostic perspective, CD5, SLCO1B1, and CD79A have been demonstrated to have protective value in various tumors, whereas ETV5, FZD7, SNX7, and SLC1A7 are involved in tumor progression. The key gene, SLCO1B1, encodes a transporter protein located on the cell membrane, which is downregulated in HCC and acts as a mediator of chemotherapeutic drugs, facilitating drug entry into cells (50, 51). In our study, we observed that the expression of SLCO1B1 decreased at the mRNA and protein levels in HCC tissues and was positively correlated with the infiltration of M1 macrophages. Furthermore, we found that SLCO1B1 overexpression inhibited the proliferation, migration, and invasion of HCC cells.

The liver is a vital immune organ containing a wide variety of immune cells. These immune cells play crucial roles in promoting tumor growth and inhibiting cancer progression. Therefore, immunotherapy has become a popular topic in tumor treatment. In this study, we combined the risk score with Spearman’s correlation analysis of immune cells and an activity analysis of the anti-cancer response process. We found that M2 macrophages and neutrophils were highly infiltrated in the high-risk group, whereas CD8+ T cells, plasma cells, and naïve B cells showed low infiltration and a more active recruitment of neutrophils. In contrast, T-, Th1, NK killer, and dendritic cells are more actively recruited in low-risk populations.

Studies have demonstrated that the induction of interleukin 4 and interleukin 13 speeds up the proliferation and metastasis of HCC cells in M2 macrophages (52, 53). Additionally, neutrophils play an essential immunosuppressive role in the tumor microenvironment, promote tumor progression, and serve as prospective treatment targets for HCC (54). However, CD8+ T cells mainly mediate tumor cell killing and infiltrate at lower levels into the tumor microenvironment of HCC (55). Furthermore, naïve B cells, which are the main immune cells involved in adaptive immunity and assist other immune cells in their anti-cancer role, have a reduced relative proportion in HCC (56). Patients in the low-risk group had higher immune scores and significantly more expressed immune checkpoint-related genes than those in the high-risk group, indicating that they may be more sensitive to immunotherapy. Moreover, we found that IFN-γ signaling was significantly enriched in low-risk populations. IFN-γ acts as an anti-tumor factor and plays an immunosuppressive role in tumors such as melanoma and lung cancer by enhancing the immune response of T lymphocytes (57). However, the p53 signaling pathway, cell cycle, microRNAs in cancer, and DNA replication were significantly enriched in the high-risk populations. In our constructed signature, the risk score was consistent with the expression levels of tumor-infiltrating immune cells and their immune checkpoints, indicating that the high-risk group had a stronger immunosuppressive microenvironment that promoted tumorigenesis and metastasis, leading to a worse prognosis. Future studies on immune checkpoints may benefit low-risk groups expected to have a better prognosis.

Our study provides a new direction for personalized targeted therapy in patients with HCC. However, this study has several limitations. First, all patient information was obtained from public databases and previous surgical patients at our hospital, which lacked representative prospective data. Secondly, the clinical information of the samples was limited, and some essential factors for determining patient prognosis, such as alpha-fetoprotein, ascites, portal hypertension, and postoperative complications, were missing. in the future, we plan to recruit more patients who meet our criteria at our hospital for prospective research and improve mechanistic research to gain an in-depth understanding of the clinical application value of this signature.

## 5 Conclusions

Recently, bioinformatics has become increasingly popular in the medical field. Benefiting from progress in this technology, we developed a prognostic signature for HCC based on disulfidptosis and GRGs. The signature showed a strong performance in predicting patient prognosis and response to immunotherapy, among other factors. In the future, it will have broad application prospects in the treatment of HCC. It can identify high-risk patients early and screen potential patients for immunotherapy to improve their survival. In addition, we found that SLCO1B1 is an important component of this signature; the gene is under-expressed in HCC and suppresses the proliferation, migration, and invasion of HCC cells. To some extent, these findings guide the development of targeted therapies for HCC.

## Data availability statement

The datasets presented in this study can be found in online repositories. The names of the repository/repositories and accession number(s) can be found in the article/**Supplementary Material**.

## Ethics statement

The studies involving humans were approved by The Ethics Committee of the First Affiliated Hospital of Chongqing Medical University. The studies were conducted in accordance with the local legislation and institutional requirements. The participants provided their written informed consent to participate in this study.

## Author contributions

ZW designed the study. ZW and XNC analyzed the data, conducted the experiments, and drafted the manuscript. JZ, XXC, JP and WH revised the manuscript. All authors contributed to the article and approved the submitted version.
