# Correction: Based on disulfidptosis-related glycolytic genes to construct a signature for predicting prognosis and immune infiltration analysis of hepatocellular carcinoma

**DOI:** 10.3389/fimmu.2026.1780944

**Published:** 2026-01-20

**Authors:** Zhijian Wang, Xuenuo Chen, Jia Zhang, Xuanxin Chen, Jiayi Peng, Wenxiang Huang

**Affiliations:** 1 Department of General Practice, The First Affiliated Hospital of Chongqing Medical University, Chongqing, China; 2 Department of Infectious Disease, The First Affiliated Hospital of Chongqing Medical University, Chongqing, China; 3 Department of Geriatrics, The First Affiliated Hospital of Chongqing Medical University, Chongqing, China

**Keywords:** hepatocellular carcinoma (HCC), disulfidptosis, glycolysis, subtype, prognostic signature, tumor microenvironment, SLCO1B1

In the published article, there was an error in **Figure 11** as published. The corrected **Figure 11** and its caption “Correlation of SLCO1B1 expression with the level of infiltration of various immune cells in cancers”. appear below.

**Figure 11:**
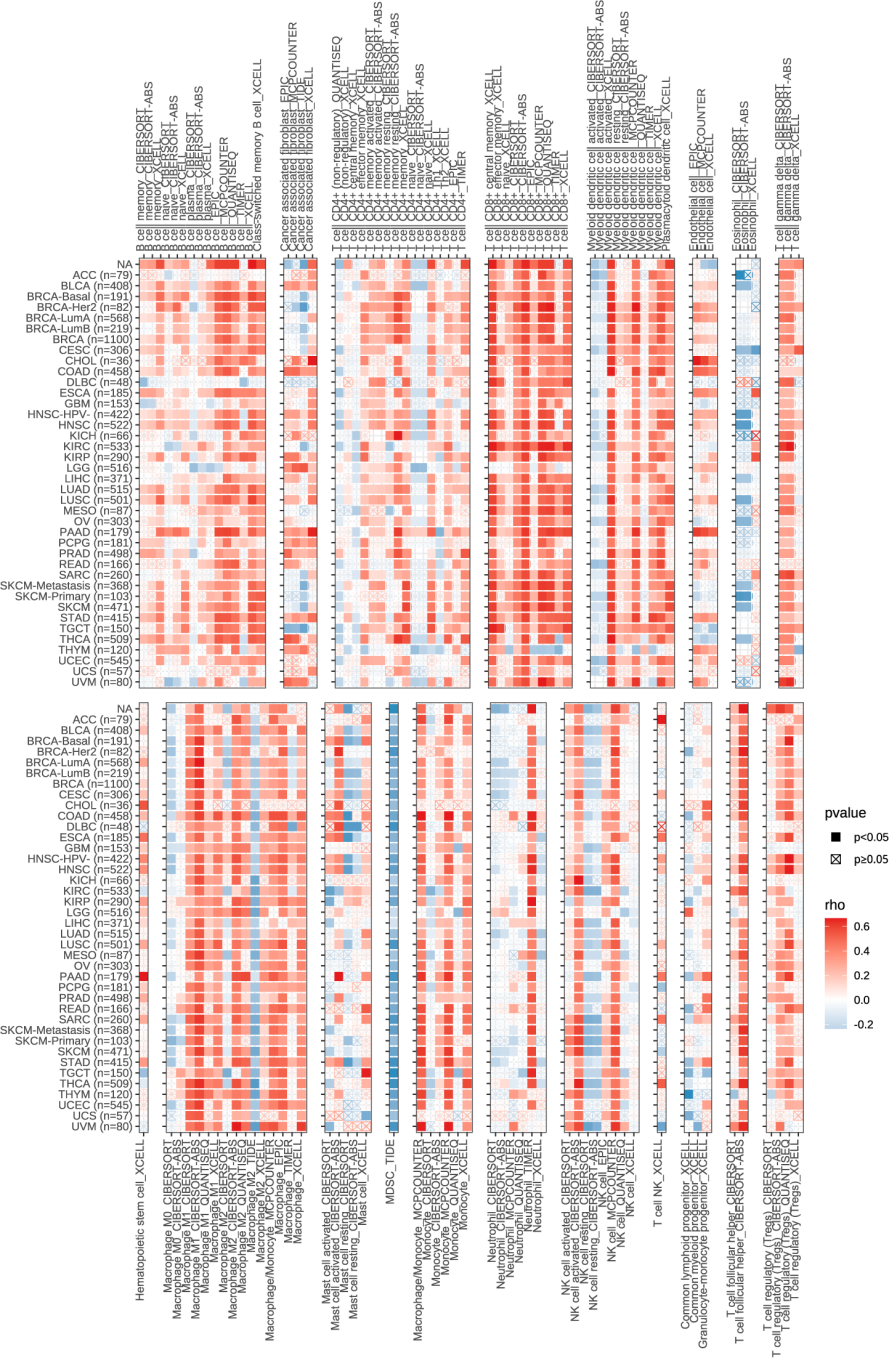
Correlation of SLCO1B1 expression with the level of infiltration of various immune cells in cancers.

The authors apologize for this error and state that this does not change the scientific conclusions of the article in any way. The original article has been updated.

