# Supplementary material for: Based on disulfidptosis-related glycolytic genes to construct a signature for predicting prognosis and immune infiltration analysis of hepatocellular carcinoma
Source: Front Immunol. 2023 Aug 23;14:1204338. doi: 10.3389/fimmu.2023.1204338 (PMC10482091; doi:10.3389/fimmu.2023.1204338)
Supplement: Supplementary file 1 [file DataSheet_1.docx]

Supplementary Material

Construction of a Prognostic Signature for Hepatocellular Carcinoma Based on Disulfidptosis-Related Glycolytic Genes, and Immune Infiltration Analysis

Zhijian Wang, Xuenuo Chen, Jia Zhang, Xuanxin Chen, Jiayi Peng, Wenxiang Huang*

*** Correspondence:** Wenxiang Huang: [wenxianghuang2018@163.com](mailto:wenxianghuang2018@163.com)

Jianguoyun/Nutstore：<https://www.jianguoyun.com/p/DclC50YQv6XLCxi64IIFIAA>

## Supplementary Figures

**
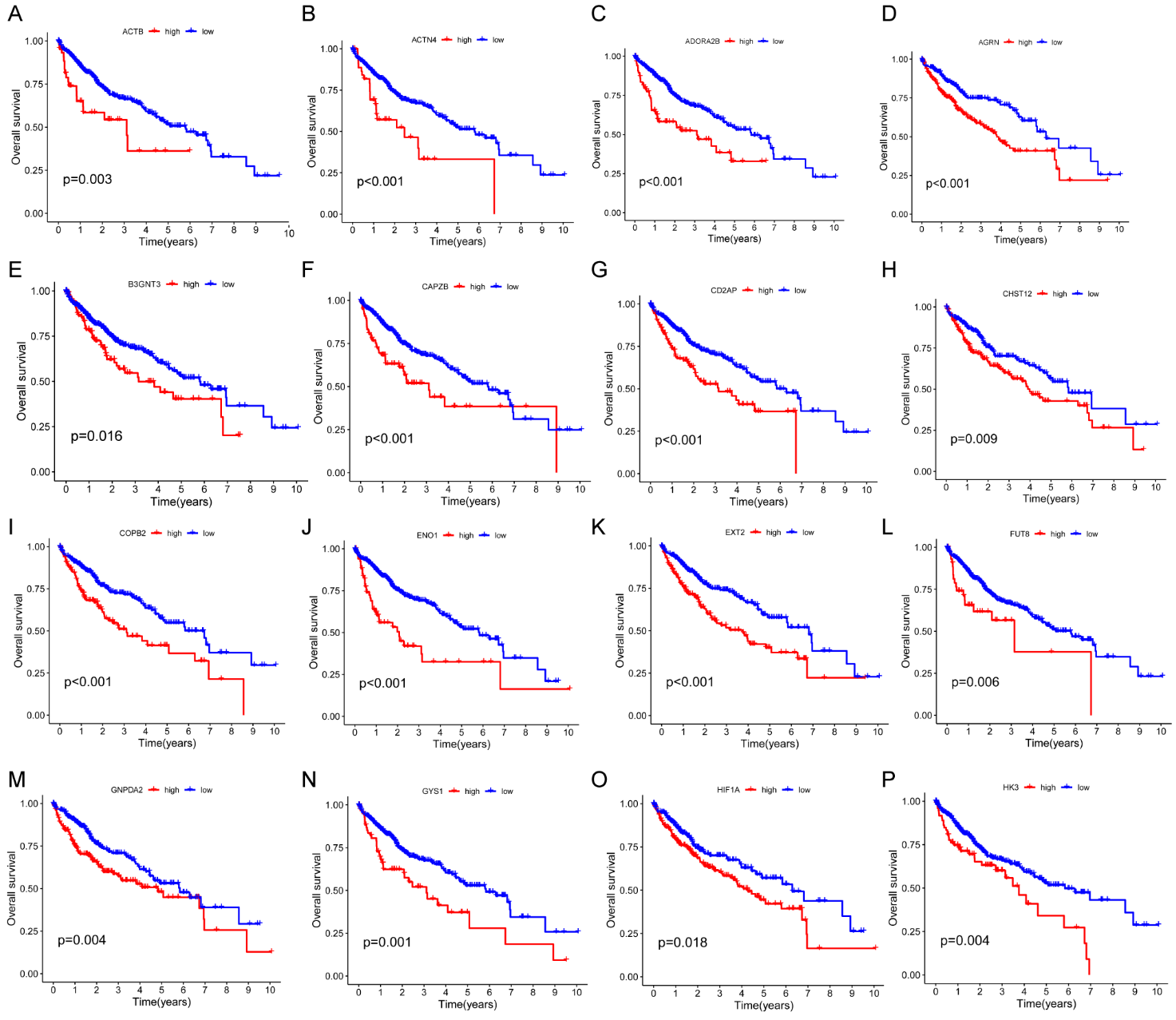
**

**Supplementary Figure 1.** (A-P) The Kaplan–Meier curves for survival status and survival time in the high and low expression groups of DRGGs. (A) ACTB. (B) ACTN4. (C) ADORA2B. (D) AGRN. (E) B3GNT3. (F) CAPZB. (G) CD2AP. (H) CHST12. (I) COPB2. (J) ENO1. (K) EXT2. (L) FUT8. (M) GNPDA2. (N) GYS1. (O) HIF1A. (P) HK3.


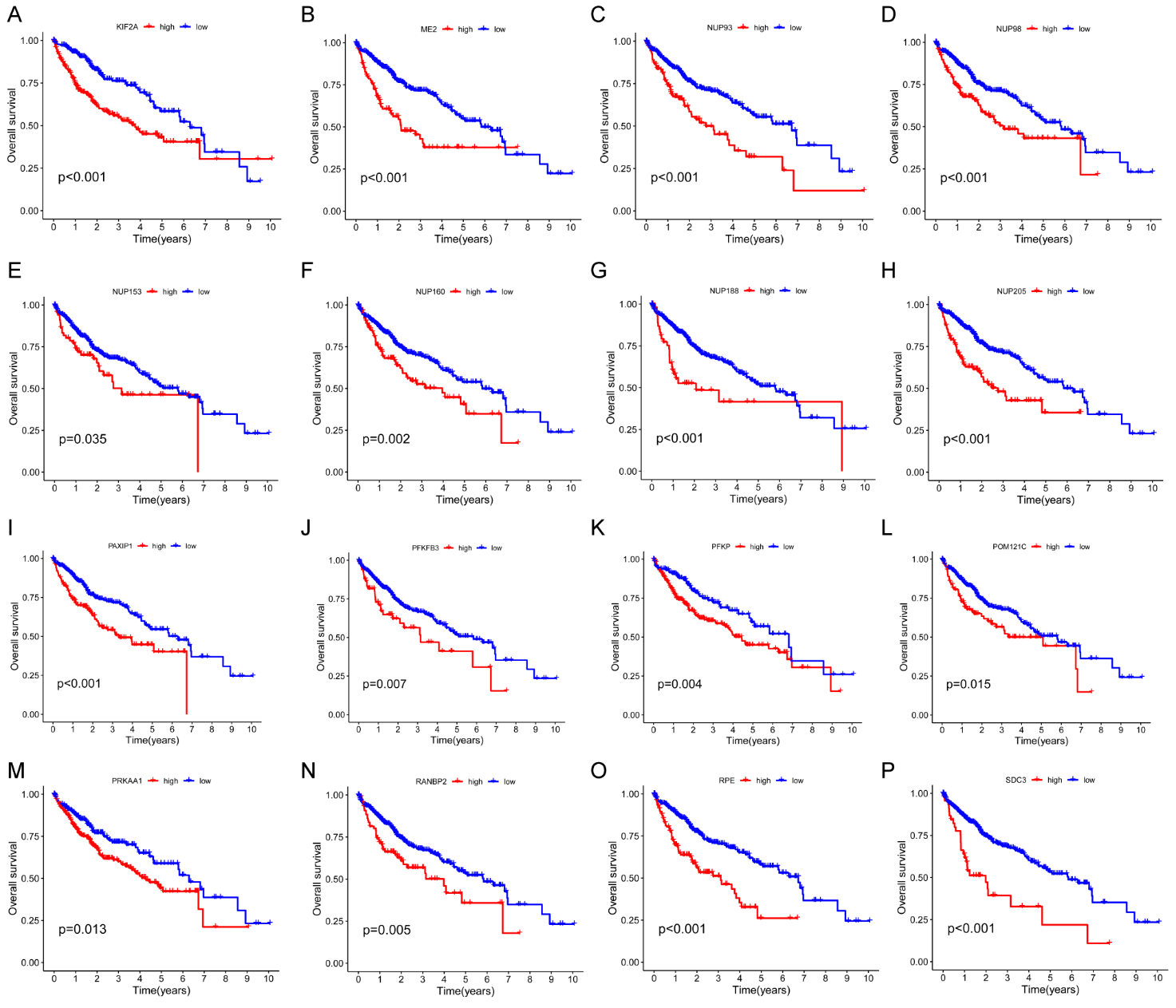


**Supplementary Figure 1.** (A-P) The Kaplan–Meier curves for survival status and survival time in the high and low expression groups of DRGGs. (A) KIF2A. (B) ME2. (C) NUP93. (D) NUP98. (E) NUP153. (F) NUP160. (G) NUP188. (H) NUP205. (I) PAXIP1. (J) PFKFB3. (K) PFKP. (L) POM121C. (M) PRKAA1. (N) RANBP2. (O) RPE. (P) SDC3.

**
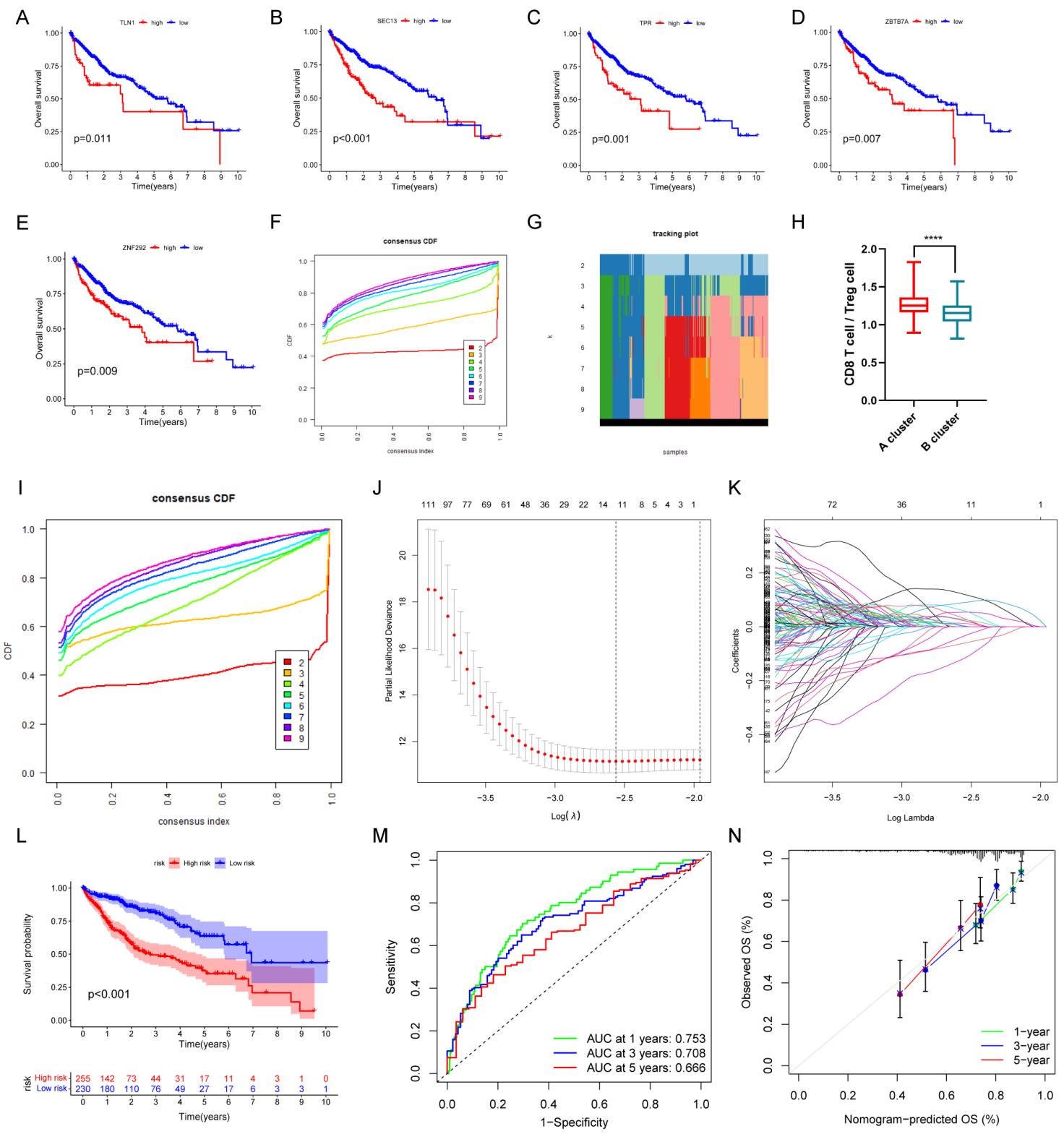
**

**Supplementary Figure 3.** (A-E) The Kaplan–Meier curves for survival status and survival time in the high and low expression groups of DRGGs. (A) TLN1. (B) SEC13. (C) TPR. (D) ZBTB7A. (E) ZNF292. (F) Cumulative density function (CDF) curves validate the clustering accuracy. (G) The tracking plot validates the clustering accuracy. (H) The ratio of CD8 T cells/Tregs in different DRGGs clusters. (I) CDF curves validate the clustering accuracy. (J-K) LASSO regression analysis and consistency test. (L) The Kaplan–Meier curves for survival status and survival time of all patients in the high-risk and low-risk groups. (M) ROC curves for predicting the 1-, 3-, and 5-year survival rates of all patients. (N) Calibration plot of the nomogram.
