# Supplementary material for: Based on disulfidptosis-related glycolytic genes to construct a signature for predicting prognosis and immune infiltration analysis of hepatocellular carcinoma
Source: Front Immunol. 2023 Aug 23;14:1204338. doi: 10.3389/fimmu.2023.1204338 (PMC10482091; doi:10.3389/fimmu.2023.1204338)
Supplement: Supplementary file 8 [file Table_7.docx]

Supplementary Table 7. Statistical results of immunohistochemistry for SLCO1B1 protein expression in HCC tissure and normal liver tissues.

|  | **Tissue** | | **χ2**  **P value** |
| --- | --- | --- | --- |
|  | **Normal (n=18)** | **Cancer (n=18)** |  |
| **Immunohistochemical grade** | 16(88.89%)  2(11.11%) | 2(11.11%)  16(88.89%) | 21.778  ＜0.0001 |
| High expression of SLCO1B1  Low expression of SLCO1B1 |  |  |  |
